# Fabricating Antibacterial and Antioxidant Electrospun Hydrophilic Polyacrylonitrile Nanofibers Loaded with AgNPs by Lignin-Induced In-Situ Method

**DOI:** 10.3390/polym13050748

**Published:** 2021-02-28

**Authors:** Md. Kaiser Haider, Azeem Ullah, Muhammad Nauman Sarwar, Takumi Yamaguchi, Qianyu Wang, Sana Ullah, Soyoung Park, Ick Soo Kim

**Affiliations:** 1 Nano Fusion Technology Research Group, Institute for Fiber Engineering (IFES), Interdisciplinary Cluster for Cutting Edge Research (ICCER), Shinshu University, Tokida 3-15-1, Ueda, Nagano 386-8567, Japan; kaisershakil@yahoo.com (M.K.H.); 08tex101@gmail.com (A.U.); nsoctober5@gmail.com (M.N.S.); 20fs325k@shinshu-u.ac.jp (T.Y.); 19fs304a@shinshu-u.ac.jp (Q.W.); sanamalik269@gmail.com (S.U.); 2 Department of Chemistry, Graduate School of Science, Kyoto University, Kitashirakawa-oiwakecho, Sakyo-ku, Kyoto 606-8502, Japan; soyoung.3z@kyoto-u.ac.jp

**Keywords:** lignin, silver nanoparticles, antibacterial activity, silver composite membranes

## Abstract

Concerning the environmental hazards owing to the chemical-based synthesis of silver nanoparticles (AgNPs), this study aimed to investigate the possibility of synthesizing AgNPs on the surface of polyacrylonitrile (PAN) nanofibers utilizing biomacromolecule lignin. SEM observations revealed that the average diameters of the produced nanofibers were slightly increased from ~512 nm to ~673 nm due to several factors like-swellings that happened during the salt treatment process, surface-bound lignin, and the presence of AgNPs. The presence of AgNPs was validated by transmission electron microscope (TEM) and X-ray photoelectron spectroscopy (XPS) analysis. The amount of synthesized AgNPs on PAN nanofibers was found to be dependent on both precursor silver salt and reductant lignin concentration. Fourier transform infrared-attenuated total reflectance (FTIR-ATR) spectra confirm the presence of lignin on PAN nanofibers. Although the X-ray diffraction pattern did not show any AgNPs band, the reduced intensity of the stabilized PAN characteristics bands at 2θ = 17.28° and 29.38° demonstrated some misalignment of PAN polymeric chains. The water contact angle (WCA) of hydrophobic PAN nanofibers was reduced from 112.6 ± 4.16° to 21.4 ± 5.03° for the maximum AgNPs coated specimen. The prepared membranes exhibited low thermal stability and good swelling capacity up to 20.1 ± 0.92 g/g and 18.05 ± 0.68 g/g in distilled water and 0.9 wt% NaCl solution, respectively. Coated lignin imparts antioxidant activity up to 78.37 ± 0.12% at 12 h of incubation. The resultant nanofibrous membranes showed a proportional increase in antibacterial efficacy with the rise in AgNPs loading against both Gram-positive *S. aureus* and Gram-negative *E. coli* bacterial strains by disc diffusion test (AATCC 147-1998). Halos for maximum AgNPs loading was calculated to 18.89 ± 0.15 mm for *S. aureus* and 21.38 ± 0.17 mm for *E. coli*. An initial burst release of silver elements within 24 h was observed in the inductively coupled plasma-atomic emission spectrometry (ICP-AES) test, and the release amounts were proportionally expansive with the increase in Ag contents. Our results demonstrated that such types of composite nanofibers have a strong potential to be used in biomedicine.

## 1. Introduction

Electrospinning is a simple, cheap, and versatile technique for producing multifunctional nanofibers from a wide variety of materials that include polymers, polymer blends, sol-gels, ceramics, composites, etc. [1,2]. Nanofibers are defined as structures with a diameter of less than 1 μm by the U.S. textile industry and Japanese and Korean strategic research initiatives, which is different from the National Science Foundation definition of nanotechnology, where the structure less than 0.1 μm are classified as nanomaterials [3]. Nanowebs from electrospun nanofibers have remarkable uniqueness, such as a large surface-to-volume ratio and pore sizes in the nano range. Additionally, modifying nanofibers’ functionality is much easier because of the flexibility of incorporating different additives on the solution during the electrospinning process [1,2,4,5]. Electrospun nanofibers’ functionality can also be enhanced by surface anchoring of various functional elements. The exclusive properties and multi-functionality of these nanowebs make them extremely interesting and appealing for applications in multiple areas, including biotechnology and nanotechnology, with particular applications in areas like membranes/filtration, textiles, sensors, medical scaffolds, etc. [6,7,8,9,10,11].

Nanofibers, surface-functionalized with AgNPs, have gained attention as an efficient antimicrobial material [12,13]. Silver is a widely used and broad-spectrum biocidal agent that is effective against fungi and bacteria but shows less cytotoxicity to human cells [14,15,16,17]. Rujitanaroj et al. prepared gelatin AgNPs composite nanofibers that exhibited superior antibacterial activity against several wound infecting bacterial strains [4]. Kharaghani et al. fabricated AgNPs-functionalized PAN nanofibers as a promising disinfecting membrane filter. Their prepared nanocomposites showed appreciable antibacterial efficacy against *Escherichia coli (E. coli)* and *Staphylococcus aureus (S. aureus)* bacteria. Further, their prepared membranes revealed good biocompatibility with mammalian cells in an adequate AgNPs loading range [18]. A comparative study by Castellano et al. revealed the antibacterial activity of Ag-containing commercial dressings. Their results demonstrated that although those Ag-containing dressings exhibited good antibacterial activity, their efficiency is lower than that of topical antimicrobial agents [19]. The most commonly used method for combining AgNPs with electrospun nanofibers is by directly mixing AgNPs into the pre-electrospinning polymer solutions [20,21]. However, nanofibers produced using this doping method have demonstrated diminished antimicrobial efficiency due to AgNPs aggregation and subsequently reduced bioavailability. Previous research has shown that synthesizing nanoparticles through immersion techniques on electrospun nanofibers is more efficient than the doping or solution reduction process [22,23]. In addition to research on different AgNPs synthesizing techniques on electrospun nanofibers, the effectiveness of varying silver salt reducing agents was also studied by several researchers. 

Reduction of silver nitrate (AgNO_3_) in polymeric solutions by hydrazinium hydroxide [24], borohydride [25], hydrogen gas [13], citrate [26], ascorbate [27], etc., have been reported. Environmental concern leads the researchers to avoid using environmentally hazardous and dangerous chemical agents as a silver salt reducing agent. Thus, several green methods for AgNPs synthesis using plant extracts were investigated [28,29,30,31,32,33]. Synthesis of AgNPs using UV radiation [34], gamma radiation [35], microwave [36], and laser irradiation pulse [37] was also studied by several researchers in recent years. However, these reports focused on the synthesis of AgNPs only rather than synthesis on electrospun nanofibrous structures. These green methods typically require longer treatment times and lengthy AgNPs synthesis procedures, making them prodigal and nonviable for practical applications.

Lignin is an underutilized amorphous polyphenolic biomacromolecule comprising several polar functional groups, i.e., thiols, reductive aliphatic, and phenolic hydroxyls. These groups’ presence on lignin structure makes it a suitable candidate for reducing and stabilizing metallic nanoparticles. Although the use of lignin in carbon nanofiber production has been reported by several researchers [38,39,40,41,42], reports on nanoparticle synthesis on electrospun nanofiber using lignin are limited [43]. Several reports on the synthesis of AgNPs using different sources/types of lignin can be found in recent literature demonstrating the potentiality of lignin as a metal reducing agent [44,45,46,47,48,49]. Only one research report on the synthesis of AgNPs on electrospun cellulose nanofibers using alkali lignin was found in the literature [50]. In addition, the reducing capability of different concentrations of lignin to synthesize AgNPs on the nanofibrous structure and their possible effects on the base polymer are needed to be explored.

Chemical-induced reduction of AgNPs is always associated with environmental pollution. However, utilizing biological macromolecule lignin is advantageous in terms of availability, environmentally friendly, uses safety, and cost-efficiency. Furthermore, antioxidant lignin-induced AgNPs coating on PAN nanofibers offers highly efficient functional antibacterial applications. Hence, we designed our research on synthesizing AgNPs on the surface of electrospun PAN nanofibers using alkali lignin as a reducing and binding agent. We used three different concentrations of AgNO_3_ as a metal precursor, and each AgNO_3_ treated sample was studied against two different concentrations of alkali lignin. Thus, we can investigate the influence of alkali lignin concentration on AgNPs synthesis. Nanofibrous morphology, chemical interactions, structural changes in PAN polymer, thermal stability, and surface wetting behavior were depicted through different characterization tools. Two most common bacterial strains, namely—Gram-negative *Escherichia coli (E. coli)* and Gram-positive *Staphylococcus aureus (S. aureus)* were selected to evaluate the antibacterial efficacy of the prepared membranes. The Ag release kinetics, excellent antibacterial performance, and lignin-derived antioxidant activity demonstrated that the prepared nanofibrous membranes have strong potential to be used in biomedicine and other antibacterial applications. 

## 2. Materials and Methods

### 2.1. Materials

PAN powder (Mw = 150,000 g/mol) and Alkali lignin (low sulfonate content: ≤3.6%) were purchased from Sigma-Aldrich, Tokyo, Japan. N,N-Dimethylformamide (DMF 99.8%) and silver nitrate (AgNO_3_) were purchased from Wako Pure Chemical Industries, Osaka, Japan. DPPH (1, 1-diphenyl-2-picryl hydrazyl radical) was purchased from Tokyo Chemical Industry Co., Ltd, Japan. Distilled water was used from the laboratory water distillation plant. All chemicals were used without any further purification.

### 2.2. Methods

#### 2.2.1. Fabrication of PAN Nanofibers via Electrospinning 

Firstly, 10% by weight PAN powder was thoroughly dissolved in DMF to prepare polymer solution for electrospinning. The solution was vigorously stirred for 24 h under room temperature to ensure all components were mixed properly. The electrospinning apparatus was set up with a high voltage power supply (Har-100*12, Matsusada Co., Tokyo, Japan), a syringe pump, and a rotating/reciprocating grounded collector. A 20 mL plastic syringe, with a metallic needle (22 G) affixed to it, was used to contain the prepared solution. The needle’s inner diameter was 0.4 mm, and the TCD (distance between the tip of the needle and the collector) was adjusted to 15 cm. The syringe pump was set to a flow rate of 0.7 mL h^−1^, and the spinning was performed for 48 h to obtain a uniform PAN nanofibrous membrane. The electrospinning parameters were set at a voltage of 18 kV, room temperature, and ~40% humidity. All nanofibrous samples were stabilized under air atmosphere for 2 h at 250 °C temperatures at a heating and cooling rate of 10 °C/min.

#### 2.2.2. Synthesis of AgNPs on the Surface of PAN Nanofibers

The process of synthesizing AgNPs on PAN nanofibers’ surface involved immersing 1 gm PAN nanofibrous membrane into 50 mL of 5mM, 10mM, and 15 mM AgNO_3_ solution under gentle shaking, 300 rpm for 24 h as a first step. After fast rinsing with distilled water, the nanofiber membranes were again immersed into 2 g/L and 4 g/L alkali lignin solution (50 mL) under gentle shaking, 300 rpm for 24 h. Finally, all nanofiber samples were washed thoroughly with distilled water until the final rinse was clear and dried in a laboratory oven at 60 °C temperatures for 24 h to obtain PAN nanofibers surface-functionalized with AgNPs (PAN/AgNPs). The coding of PAN/AgNPs is illustrated in Table 1.

### 2.3. Characterizations

#### 2.3.1. Morphological Analysis

Scanning Electron Microscope (SEM) (JSM-5300, JEOL Ltd., Tokyo, Japan) with 10 kV acceleration voltage and Transmission Electron microscope (TEM) (JEOL 2010 Fas TEM, Tokyo, Japan) with 200 kV accelerating voltage was utilized to investigate the morphological properties of PAN and PAN/AgNPs. All the samples were sputtered coated with platinum (Pt) for 160s at 20mA before SEM analysis. The average diameter of the PAN and PAN/AgNPs was measured by analyzing the SEM images employing the ImageJ 1.50i software. Excel and results were displayed in mean values with standard deviations. This software was also used to measure the nanoparticle size by analyzing the TEM images. The nanofibers’ average diameter was measured over 50 nanofiber strands for each sample, and the size of AgNPs was measured by sampling over 50 nanoparticles.

#### 2.3.2. Physicochemical Analysis

Fourier Transform Infrared-Attenuated Total Reflectance (FTIR-ATR) (Prestige-21, Shimadzu Co., Ltd., Tokyo, Japan) Spectroscopic analysis between the wavenumber range of 600 to 4000 cm^−1^ was employed to determine the influence of produced nanoparticles on the chemical structure of PAN nanofibers.

#### 2.3.3. WAXRD Analysis

The influence of AgNPs toward fibrous crystallinity was investigated by Wide Angle X-ray diffraction instrument (WAXRD) with angular ranging 5° ≤ 2θ ≤ 90°, equipped with Nickel-filtered CuKα (Rotaflex RT300 mA, Rigaku Co., Osaka, Japan).

#### 2.3.4. Elemental Analysis

Elemental analysis was studied by X-ray Photoelectron Spectroscopy (XPS) (Kratos Axis-Ultra DLD, Kratos Analytical, Manchester, UK).

#### 2.3.5. Surface Wetting Properties 

The surface wetting properties of the produced nanofibrous membranes were evaluated by water contact angle test, at 24% relative humidity and 17 °C temperature using a contact angle meter (Digidrop, GBX France). A syringe with a 22 G needle containing 5 microliters of freshly prepared distilled water was used for this test.

#### 2.3.6. Swelling Ratio and Yield Measurements of PAN/AgNPs

The swelling ratio of PAN/AgNPs was measured by immersing 5 mg of each sample into 10 mL distilled water for 24 h at ambient temperature until the PAN/AgNPs had reached the equilibrium state of swelling through absorption of water into the nanofibrous network. This was aimed to simulate the swelling process in vivo. The swollen nanofibrous membranes were removed from the water, and the excess water was wiped-off using filter paper, followed by measuring the swollen weight. A similar procedure was applied to swell PAN/AgNPs in 0.9 wt % NaCl solution instead of distilled water. To calculate the yield percentages of swollen PAN/AgNPs, all samples were dried at 60 °C for 24 h until a constant weight was obtained. The swelling ratios and yield (%) were calculated using the following Equations (1) and (2), respectively [51].

Swelling ratio (S.R.) = (W_S_ − W_d_)/W_d_ g/g (1)

Yield = (W_d_/W) × 100% (2)

where

W_S_ = Weight of swollen sample, g.
W_d_ = Dry weight of the sample after swelling and drying, g.
W = Dry weight of the sample before swelling, g.

#### 2.3.7. Thermal Analysis

Thermal stability of PAN and PAN/AgNPs was evaluated by Thermogravimetric analysis (TG-8120, Rigaku Corporation, Osaka, Japan) with a heating rate of 10 °C/min. Thermal degradability was observed within the temperature range of 20 °C–500 °C under air atmosphere.

#### 2.3.8. Antibacterial Evaluation of PAN/AgNPs

Antibacterial efficacy of PAN/AgNPs was assessed against Gram-negative *E. coli* and Gram-positive *S. aureus* bacteria following the disk diffusion method (AATCC 147-1998), where PAN nanofibrous membrane was used as a negative control. Round disk of samples with 10 mm diameter and weight around 0.003 g each were placed in each bacterium cultured plate and incubated for 24 h at 37 °C temperatures before measuring the inhibition zone. The zone of inhibition was measured using ImageJ 1.50i software, and the quantifying procedure was conducted based on photographs of the specimens. The diameter of the inhibition zones was presented in mm with standard deviations. 

#### 2.3.9. Ag Contents and Ag Release Kinetics 

Inductively Coupled Plasma-Atomic Emission Spectrometry (ICP-AES, Shimadzu ICPS1000 IV, Shimadzu, Kyoto, Japan) was employed to determine Ag contents and Ag release behavior of all Ag composite specimens. To measure Ag contents, samples were first immersed in concentrated HNO_3_ solution to ensure complete dissolution of Ag nanoparticles into the solution. The extracted solutions were then sampled and quantified by ICP.

The Ag release profiles of PAN/AgNPs were measured by immersing 0.02 g of each silver-containing sample in 30 mL distilled water under the gentle shaking condition, 300 rpm during the period of 2, 4, 6, 8, 10, 12 h, 1, 2, 3, 4, 5, and 6 days. To measure silver release, 2 mL of liquid specimens were extracted after a specific time and supplied to ICP-AES spectrometry.

#### 2.3.10. Antioxidant Activity

DPPH is a compound when reacted with a free radical scavenger it reduces and changes its color from purple to yellow based on the reducing efficacy of the antioxidants. To evaluate the antioxidant activity of the PAN/AgNPs membranes, 50 mg of each specimen was soaked in 10 mL 0.1 mM DPPH methanol solution and incubated for different periods (2, 6, and 12 h) at 37 °C in an incubator under a dark environment. The absorbance of the solutions at preset time intervals was monitored at 515nm wavelength using a UV-Visible spectrophotometer (Lamda 900, Perkin-Elmer, Waltham, MA, USA). The antioxidant efficiency was measured using the following Equation (3) [52,53].

(3) $$Antioxidant\;efficiency\;\left(\%\right)=\frac{An-As}{An}\times 100$$

where An and As is the absorbance of DPPH solutions without and with nanofiber membrane at 515 nm.

#### 2.3.11. Statistical Analysis

All statistical analyses were carried out using MINITAB 17 ^®^ statistical software.

## 3. Results and Discussion

### 3.1. Morphology of Nanofibers

**Figure 1:**
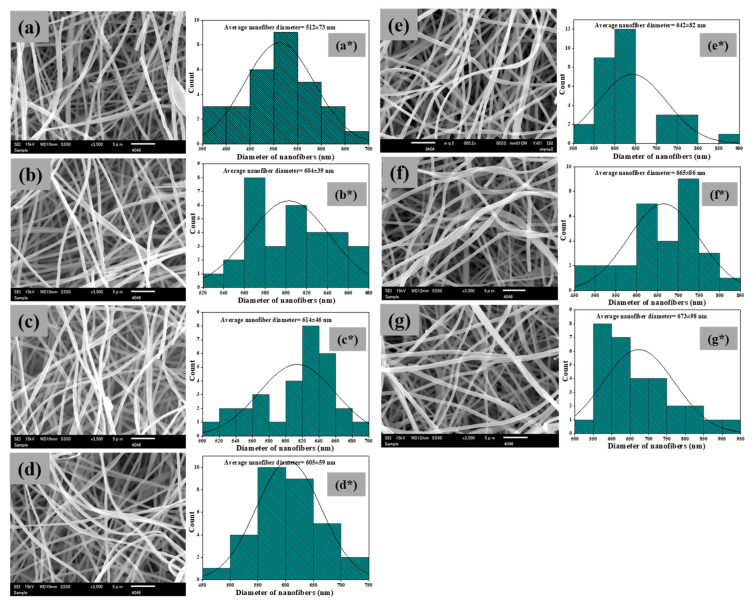
SEM images of PAN nanofiber (**a**), PAN/AgNPs1 (**b**), PAN/AgNPs2 (**c**), PAN/AgNPs3 (**d**), PAN/AgNPs4 (**e**), PAN/AgNPs5 (**f**), and PAN/AgNPs6 (**g**); nanofiber diameter distribution histograms of PAN nanofiber (a*), PAN/AgNPs1 (b*), PAN/AgNPs2 (c*), PAN/AgNPs3 (d*), PAN/AgNPs4 (e*), PAN/AgNPs5 (f*), and PAN/AgNPs6 (g*).

**Figure 2:**
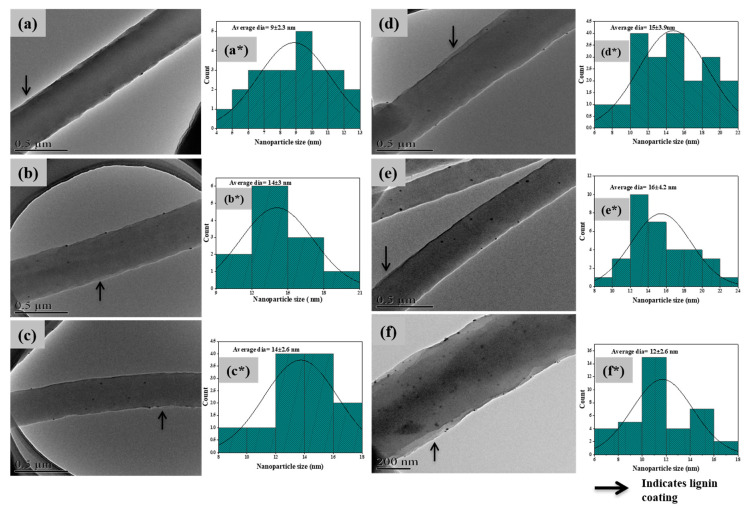
Single fiber Transmission Electron microscope (TEM) images of PAN/AgNPs1 (**a**), PAN/AgNPs2 (**b**), PAN/AgNPs3 (**c**), PAN/AgNPs4 (**d**), PAN/AgNPs5 (**e**), and PAN/AgNPs6 (**f**); nanoparticle size distribution histograms of PAN/AgNPs1 (a*), PAN/AgNPs2 (b*), PAN/AgNPs3 (c*), PAN/AgNPs4 (d*), PAN/AgNPs5 (e*), and PAN/AgNPs6 (f*).

The morphological observation reveals smooth and homogenous PAN nanofiber and PAN/AgNPs (Figure 1). The average diameter of PAN nanofibers was 512 ± 73 nm, whereas for PAN/AgNPs1, PAN/AgNPs2, PAN/AgNPs3, PAN/AgNPs4, PAN/AgNPs5, and PAN/AgNPs6, diameters were 604 ± 39, 614 ± 46, 605 ± 59, 642 ± 82, 665 ± 86, and 673 ± 98 nm, respectively. The nanofibers’ swellings during the salt treatment stage, surface-bound layer of lignin formed during lignin treatment (arrow indication in TEM images, Figure 2), and AgNPs formation contributed to the differences in mean diameters between PAN/AgNPs. 

For a closer look at nanosized silver particles, single fiber TEM images were obtained (Figure 2). In all PAN/AgNPs, homogenous distribution of AgNPs without clogging can be observed. The mean nanoparticle size with standard deviation of PAN/AgNPs1, PAN/AgNPs2, PAN/AgNPs3, PAN/AgNPs4, PAN/AgNPs5, and PAN/AgNPs6 were 9 ± 2.3, 14 ± 3.0, 14 ± 2.6, 15 ± 3.9, 16 ± 4.2, and 12 ± 2.6 nm, respectively. However, for the low concentration of silver salts (5 and 10 mM), less nanoparticles were produced in PAN/AgNPs1, PAN/AgNPs2, PAN/AgNPs3, and PAN/AgNPs4 composite samples. In PAN/AgNPs5 and PAN/AgNPs6, a higher amount of nanoparticles can be observed. Although the same silver concentration was used in the later PAN/AgNPs composite samples, higher alkali lignin concentration contributed to the increase in silver salt reduction, thus increases the nanoparticle formation. The thickness of the prepared membranes, the average diameter of nanofibers, and the mean diameter of AgNPs are shown in Table 2. 

### 3.2. FT-IR Spectral Analysis

**Figure 3:**
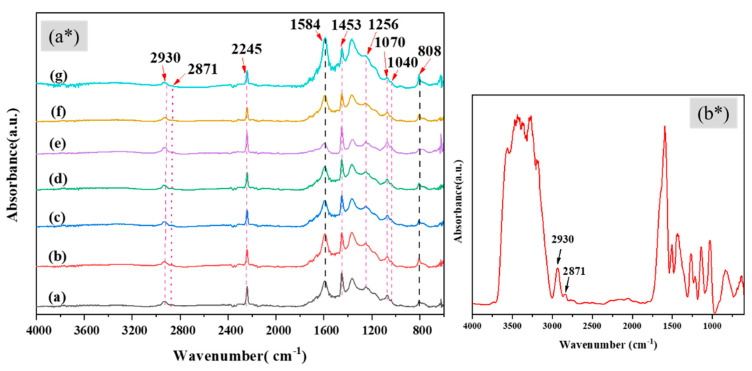
Fourier transform infrared (FTIR) spectra of (a) PAN nanofiber, (b) PAN/AgNPs1, (c) PAN/AgNPs2, (d) PAN/AgNPs3, (e) PAN/AgNPs4, (f) PAN/AgNPs5, and (g) PAN/AgNPs6 (a*); FTIR spectra of alkali lignin only (b*).

ATR-FTIR spectra of PAN and PAN/AgNPs are shown in Figure 3a*. The distinctive absorption peaks for PAN were present in all PAN/AgNPs, including C≡N stretching at 2245 cm⁻^1^, CH_2_ bending at 1453 cm⁻^1^, CH wagging at 1256 cm⁻^1^, skeletal vibration of PAN molecular chain at 1070 cm^−^^1^, and C–C≡N combination mode at 1040 cm^−^^1^ [54,55,56]. The peaks at 2930 and 2871 cm^−1^ were ascribed to CH_2_ stretching of both PAN and lignin molecules as evident from the spectra of lignin only, Figure 3b*. In addition, new absorption peaks in all PAN/AgNPs and PAN at 1584 cm^−^^1^ attributed to the combination of C=N and C=C stretching and 808 cm^−^^1^ due to C=C–H bending were present. The presence of these absorption peaks verified the successful stabilization of PAN nanofibers [57,58,59]. These absorption peaks resulted from the generations of conjugated C=C structures from hydrogenations and conjugated C=N containing structures from the nitrile groups [59,60,61,62]. A few differences can be observed in the ATR spectrum of PAN/AgNPs. The peak at 2930 cm^−^^1^ for CH_2_ asymmetric stretching showed slight positive shifting in all PAN/AgNPs, which may be the effect of newly formed Ag nanoparticles on PAN nanofibers. Chemical interactions and the changes in the packing density of the polymer molecules cause peak shifting of specific bands in infrared spectroscopy [61]. As AgNPs (zero-valent silver) have no chemical interactions with either PAN or lignin molecule, we assumed that while treating PAN with precursor AgNO_3_ salt during the particle synthesis process, interactions happened between Ag ions and asymmetric CH_2_ of PAN. Although those interactions were diminished after lignin-induced reduction of Ag^+^ to Ag^0^. Such interactions before the reduction of Ag ions into AgNPs might cause some changes in the packing density of –CH_2_, thus resulting in slight positive shifting of the corresponding band.

### 3.3. WAXRD Analysis

**Figure 4:**
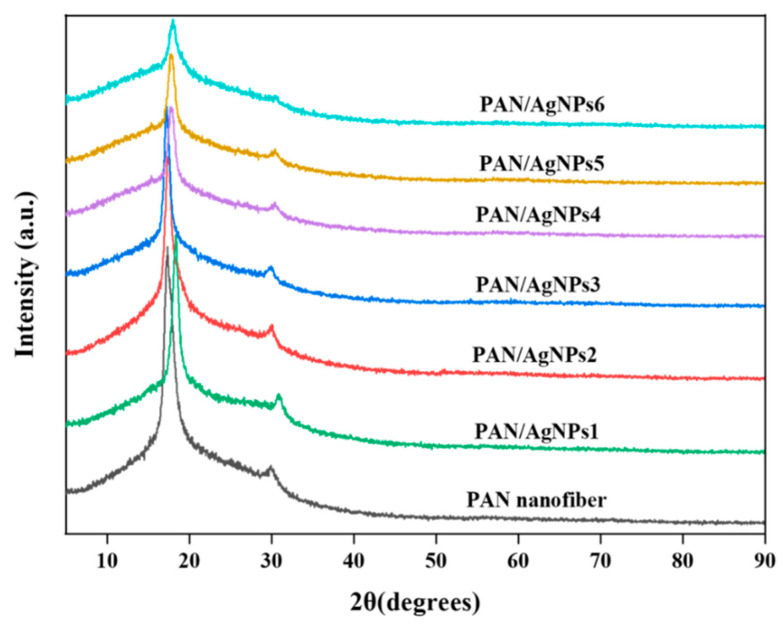
X-ray diffraction (XRD) spectra of PAN nanofiber, PAN/AgNPs1, PAN/AgNPs2, PAN/AgNPs3, PAN/AgNPs4, PAN/AgNPs5, and PAN/AgNPs6.

The differences and variations of PAN nanofibers’ crystalline structure before and after nanoparticle synthesis reactions were investigated by WAXRD analysis. Pristine electrospun PAN nanofibers depict two typical diffraction bands centered at 2θ = 16.5° and 29.34°. These two bands represent the X-ray reflections of (100) and (110) crystallographic facets of PAN nanofibers [62]. The PAN nanofibrous membrane showed two diffraction peaks at 2θ = 17.28° and 29.38° position, Figure 4. The positive shifting of 2θ = 16.5° might be attributed to the air oxidation during stabilization in the ambient conditions. The characteristic diffraction peaks of AgNPs were not observed in PAN/AgNPs samples. This is because when a low concentration (less than 100 mM) of silver salts (AgNO_3_) is used in synthesizing AgNPs on electrospun nanofiber by immersion method, every so often, XRD peaks cannot be achieved [6]. The presence of silver species on PAN nanofibers’ surface was verified by TEM and XPS analysis (Section 3.1, 3.4). The formation AgNPs on the surface of nanofibers causes little shifting and reduction in the intensity of PAN nanofibers’ diffraction pattern. This is an indication of some deterioration in the crystalline structure of the PAN polymer backbone.

### 3.4. XPS Analysis

**Figure 5:**
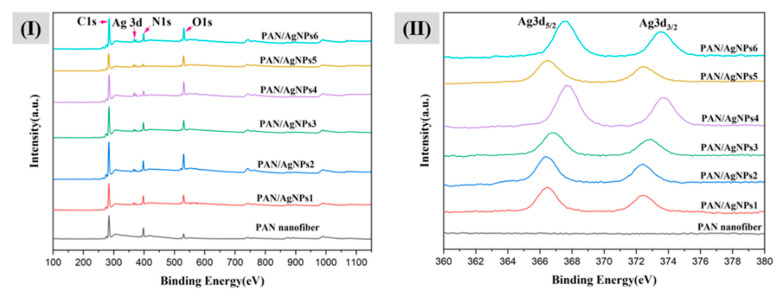
(**I**) X-ray Photoelectron Spectroscopy (XPS) wide spectra of PAN nanofiber, PAN/AgNPs1, PAN/AgNPs2, PAN/AgNPs3, PAN/AgNPs4, PAN/AgNPs5, and PAN/AgNPs6; (**II**) XPS narrow spectra of PAN nanofiber, PAN/AgNPs1, PAN/AgNPs2, PAN/AgNPs3, PAN/AgNPs4, PAN/AgNPs5, and PAN/AgNPs6 over Ag 3d region.

XPS also analyzed the contribution of the elements to PAN/AgNPs. Wide XPS surveys present C1s (284 eV), O1s (530 eV), N1s (398 eV), Ag3d (366.5 eV to 374 eV) characteristics peaks in all PAN/AgNPs, confirming their presence in all nanofibrous membranes, Figure 5I. The narrow XPS spectra in Ag 3d region of all PAN/AgNPs nanocomposite are shown in Figure 5II. The normal state of Ag 3d_5/2_ and Ag 3d_3/2_ represent binding energy of 366.5 eV and 372.5 eV (bandgap 6 eV). The positive shifting of binding energies of Ag 3d_5/2_ and Ag 3d_3/2_ can be observed in all PAN/AgNPs, demonstrating the reduction of deposited Ag^+^ to Ag^0^. To clarify this phenomenon, Yongchao et al. showed that binding energy shifts to a higher energy state when the silver ion is reduced to form metallic silver particles [63]. In addition, these two peaks showed positive shifting between PAN/AgNPs1 and PAN/AgNPs2, PAN/AgNPs3 and PAN/AgNPs4, and PAN/AgNPs5 and PAN/AgNPs6. More intense peak shifting can be observed between PAN/AgNPs3 and PAN/AgNPs4, PAN/AgNPs5 and PAN/AgNPs6. This phenomenon gives a clear indication that alkali lignin concentration has a significant effect on silver anchoring into PAN nanofibers.

### 3.5. Surface Wetting Properties

**Figure 6:**
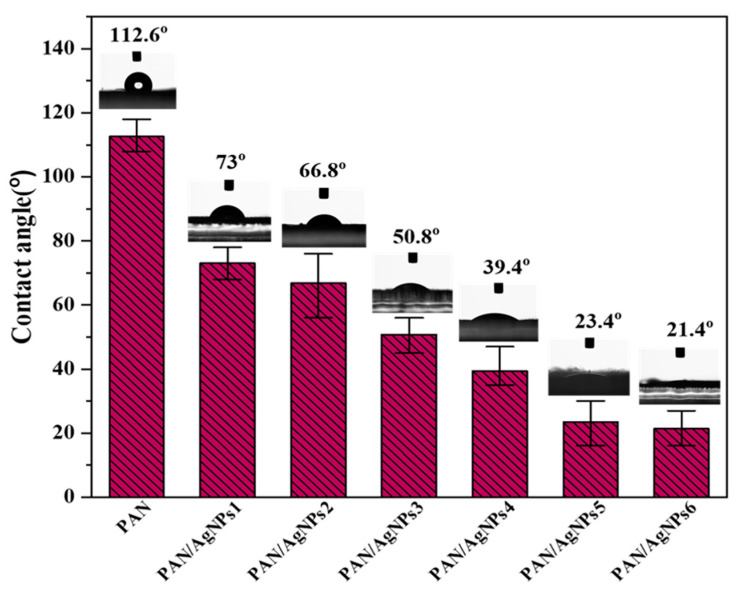
Water contact angle (WCA) of PAN nanofiber, PAN/AgNPs1, PAN/AgNPs2, PAN/AgNPs3, PAN/AgNPs4, PAN/AgNPs5, and PAN/AgNPs6.

The wettability of a material depends on various factors such as chemical composition, surface topography, etc. As the surface of any material is different from the interior, understanding the surface characteristics of materials is essential to correlate water contact properties. In the interior, atoms are in equilibrium, and interatomic forces between adjacent atoms in the crystal structure are in good balance. This is not possible at the surface because there are no interatomic interactions on the atoms’ external surface. Thus, an energy difference is created between an atom on the surface and an atom in the interior, which is called surface free energy. The decrease in water contact angle (WCA) of any material is attributed to increased surface free energy and roughness of the material [64]. The high WCA of 112.6 ± 4.16° must be due to PAN nanofibers’ hydrophobic nature. The hydrophobicity was evident from the emergence of spherically shaped water droplets on its surface for a long period. The incorporation of AgNPs on PAN has shown a significant (*P*-value = 0.000, R^2^ = 99.92) steady decrease in WCA in PAN/AgNPs, contributing towards hydrophilicity, as illustrated in Figure 6. The water contact angle of PAN/AgNPs1 is 73 ± 4.12°, PAN/AgNPs2 is 66.8 ± 7.79°, PAN/AgNPs3 is 50.8 ± 4.15°, PAN/AgNPs4 is 39.4 ± 5.13°, PAN/AgNPs5 is 23.4 ± 6.31°, PAN/AgNPs6 is 21.4 ± 5.03°. It is well known that silver particles have large surface energy, and the decrease in the size of silver is accompanied by the rise in surface free energy and surface activity. The formation of nanosized silver particles could decrease contact angle with an increase in surface free energy, which agrees with several reported literature [65]. Furthermore, the presence of surface-bound hydrophilic lignin on PAN/AgNPs also contributed to the decrease in the contact angle of all PAN/AgNPs.

### 3.6. Swelling Behavior and Yield (%)

**Figure 7:**
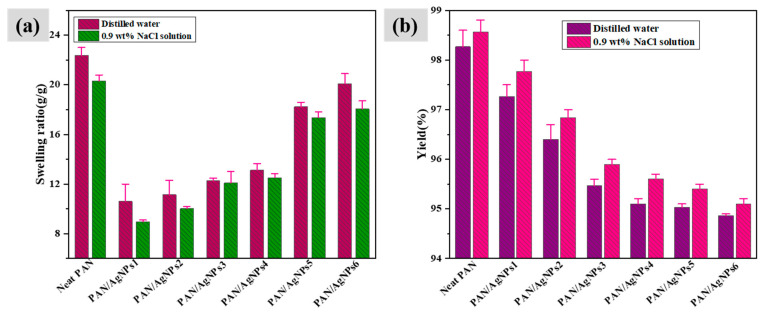
Swelling ratio (**a**) and Yield (%) (**b**) of PAN nanofiber, PAN/AgNPs1, PAN/AgNPs2, PAN/AgNPs3, PAN/AgNPs4, PAN/AgNPs5, and PAN/AgNPs6.

The liquid absorption property of polymeric nanofibers is vital while considering its practical application as wound dressing material. The swelling ratio and the yield (%) of PAN/AgNPs in distilled water and 0.9 wt % NaCl solution are shown in Figure 7a,b. The swelling ratios of neat PAN nanofibrous membrane in distilled water and 0.9 wt % NaCl solutions were 22.37 ± 0.55 and 20.3 ± 0.44 g/g, respectively, for 24 h of immersion. Although silver loaded nanofibers showed reduced swelling ratios than the neat PAN, the values proportionally increased with the silver concentration increase (*P*-value = 0.001, R^2^ = 99.62). The alkaline nature of the lignin might cause reduced inter-structure porosity on the pH-sensitive PAN nanofibers thus resulting in the lessened swelling ratios. Further, we have demonstrated form our SEM observation (Section 3.1) that the average diameters of the nanofibers of PAN/AgNPs were increased as compared to neat PAN. Such increase in the diameters of nanofibers corresponds to the reduced inter-structure porosity of the membranes. Similar observation on the effect of structural porosity on swelling capacity was found in other reported works [66]. All PAN/AgNPs samples showed lower swelling ratios in 0.9 wt % NaCl solution than distilled water. Such kind of swelling loss was often attributed to screening effect of the additional cations. The addition of excess cations in the external solution caused a non-perfect anion–anion electrostatic repulsion, which leads to a decrease in the osmotic pressure difference between the nanofibrous network and the external solution. The neat PAN nanofibrous membrane showed more than 98% yield in both distilled water and 0.9 wt % NaCl solution. Increases in the silver loading reduced the yield (%) for all PAN/AgNPs but not exceeded less than 94%. The release of silver ions and lignin from the PAN nanofiber surface into the solution might be attributed to the decrease in the yield (%) of PAN/AgNPs.

### 3.7. Thermogravimetric Analysis

**Figure 8:**
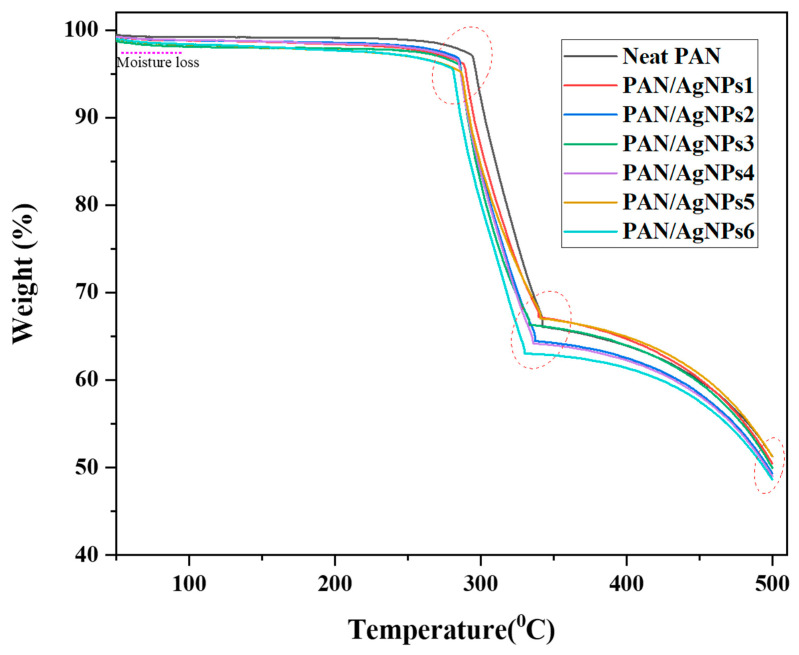
TGA analysis of PAN nanofiber, PAN/AgNPs1, PAN/AgNPs2, PAN/AgNPs3, PAN/AgNPs4, PAN/AgNPs5, and PAN/AgNPs6.

The thermal degradation behavior of stabilized neat PAN and PAN/AgNPs was evaluated using a thermogravimetric analysis using air atmosphere. A slight initial weight loss in the region 20–100 °C occurs for all nanofiber membranes, and higher moisture loss was observed for PAN/AgNPs than neat PAN nanofibers, Figure 8. In neat PAN nanofiber, major degradation occurs at 294–340 °C, which is attributed to pyrolysis of PAN polymer [67] and approximately 28% weight loss occurs between this temperature range. However, the major degradation for PAN/AgNPs1, PAN/AgNPs3, and PAN/AgNPs5 was observed at 280–330 °C and approximately 30% weight loss occurs within this temperature range. In addition, 32% weight loss occurs for PAN/AgNPs2, PAN/AgNPs4, and PAN/AgNPs6 within the same temperature range. In case of solution doped AgNPs composite nanofibers, AgNPs reduces the mobility of polymer chains, which suppress the transfer of free radicals, thus resulting in inhibiting the inter-chain interactions. Therefore, the thermal decomposition of AgNPs embedded composite nanofibers occur at higher temperatures [20]. In our work, AgNPs were surface coated on PAN nanofibers, and therefore, there is no change in the mobility of polymer chains. Thus, improvement in thermal stability was not observed in our Ag composite membranes. The residual amount of neat PAN, PAN/AgNPs1, PAN/AgNPs2, PAN/AgNPs3, PAN/AgNPs4, PAN/AgNPs5, and PAN/AgNPs6 after 500 °C was found to be 51.4%, 50.6%, 49%, 49.8%, 48.9%, 51.3%, and 48.7%, respectively.

### 3.8. Antibacterial Evaluation of PAN/AgNPs

**Figure 9:**
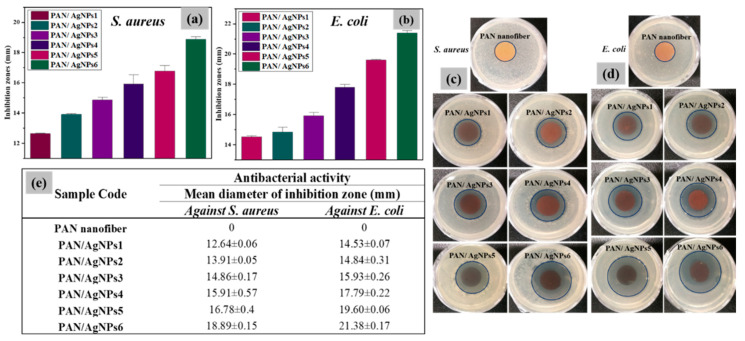
Diameters of zones of inhibition by disk diffusion test of PAN/AgNPs with standard deviations against *S. aureus* (**a**) and *E. coli* (**b**); discs showing antibacterial activity against *S. aureus* (**c**) and *E. coli* (**d**); Numerical values showing mean diameters of inhibition zones of PAN and PAN/AgNPs against *S. aureus* and *E. coli* (**e**).

Silver is proved to be the most effective among different nanosized antibacterial agents because of its highly efficient activity against bacteria, viruses, and eukaryotic micro-organisms [68]. The bactericidal activity of the PAN/AgNPs was assessed against the two most common bacterial strains, namely Gram-positive *S. aureus* and Gram-negative *E. coli*. The diameters of inhibition zones with standard deviations and the corresponding antibacterial test discs are shown in Figure 9. All Ag composite nanofibrous membranes exhibited antibacterial activity against both bacterial strains. The expansion of inhibition zones was observed with the increase in silver precursor and the reductant lignin concentration. Gram-negative bacteria are more difficult to inhibit than Gram-positive bacteria owing to their more denser cell wall structures, which make them less permeable to most antibacterial agents. Many nanofibers incorporated with AgNPs were reported to exhibit greater antibacterial efficacy against Gram-positive *S. aureus* than Gram-negative *E. coli* [18,69]. Nevertheless, in our work, clearer and larger diameter of the zone of inhibition against *E. coli* was observed compared to *S. aureus*. The better antibacterial action of AgNPs against *E. coli* than *S. aureus* was also evidenced by other scientific reports [70,71]. The diameters of zone of inhibition were calculated to 12.64 ± 0.06, 13.91 ± 0.05, 14.86 ± 0.17, 15.91 ± 0.57, 16.78 ± 0.4, and 18.89 ± 0.15 mm against *S. aureus* and 14.53 ± 0.07, 14.84 ± 0.31, 15.93 ± 0.26, 17.79 ± 0.22, 19.60 ± 0.06, and 21.38 ± 0.17 mm against *E. coli* for PAN/AgNPs1, PAN/AgNPs2, PAN/AgNPs3, PAN/AgNPs4, PAN/AgNPs5, and PAN/AgNPs6, respectively.

### 3.9. Ag Content and Ag Release Profile

**Figure 10:**
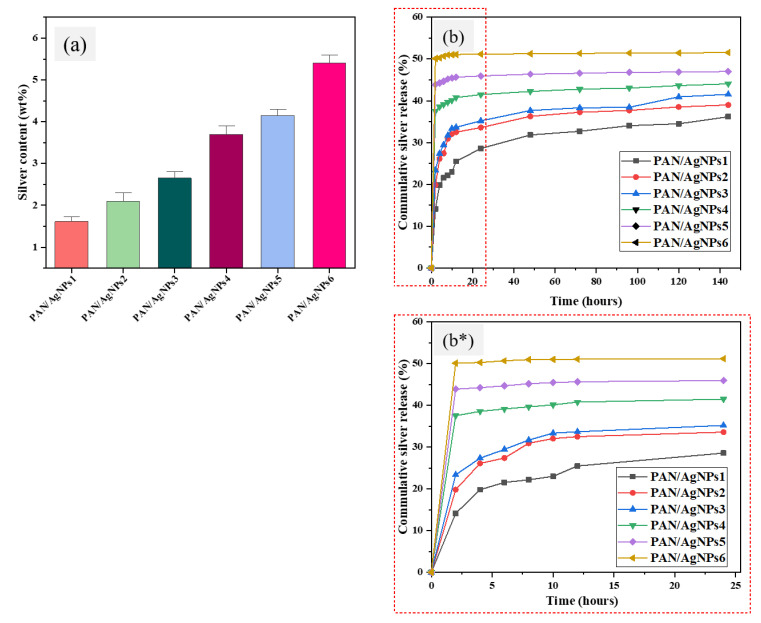
Ag contents (**a**) and Ag release kinetics of PAN/AgNPs1, PAN/AgNPs2, PAN/AgNPs3, PAN/AgNPs4, PAN/AgNPs5, and PAN/AgNPs6 for 6 days (**b**), and 24 h (b*) measured by Inductively Coupled Plasma-Atomic Emission Spectrometry (ICP-AES).

To determine Ag contents of PAN/AgNPs nanofibrous membranes, 15 mg of each specimen was immersed in 50 mL concentrated HNO_3_ solution for 24 h, and subsequently, ICP was performed on these extracted solutions to measure the Ag concentrations. Standard solutions having Ag concentrations of 5, 10, and 50 ppm were prepared for making a standard calibration curve. The coefficient factor R^2^ was found to be 0.9993. A linear correlation was found between Ag contents and the concentration of both lignin and Ag precursor solutions. The amount of Ag species present in PAN/AgNPs was calculated to 1.6 ± 0.16, 2.1 ± 0.27, 2.7 ± 0.21, 3.7 ± 0.28, 4.15 ± 0.21, and 5.4 ± 0.29 wt % for PAN/AgNPs1, PAN/AgNPs2, PAN/AgNPs3, PAN/AgNPs4, PAN/AgNPs5, and PAN/AgNPs6, respectively, Figure 10a.

As the bactericidal effects of PAN/AgNPs are due to the release of silver elements from the nanoparticles, a release kinetics study over different periods was conducted. A faster initial release and the subsequent slow-release profiles of silver elements are advantageous for different applications. A quick release of silver can be observed within 2 h of immersion, followed by a gradual increase till 24 h. A similar observation was reported in previously published works [72]. This release amount till 24 h is accounted for ~90% of total silver release, Figure 10b,b*. The release quantities of silver from all PAN/AgNPs for the next five-day course remain almost stable. A higher amount of silver released in an aqueous environment was observed for higher silver-loaded samples. The maximum amount of silver, discharged at the point of time of six days, were 36.1 ± 0.8%, 39 ± 0.4%, 41.5 ± 0.7%, 44.05 ± 0.4%, 47.02 ± 0.8%, and 51.54 ± 0.5% for PAN/AgNPs1, PAN/AgNPs2, PAN/AgNPs3, PAN/AgNPs4, PAN/AgNPs5, and PAN/AgNPs6, respectively.

### 3.10. Antioxidant Activity Analysis

**Figure 11:**
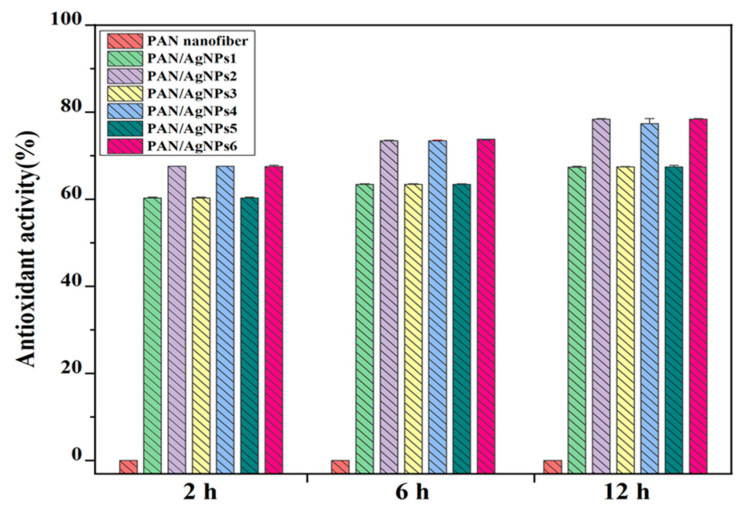
DPPH scavenging activity of PAN nanofiber, PAN/AgNPs1, PAN/AgNPs2, PAN/AgNPs3, PAN/AgNPs4, PAN/AgNPs5, and PAN/AgNPs6 measured by UV–visible spectrophotometer.

Lignin is a well-known oxygen free radical scavenger for stabilizing the reactions initiated by oxygen free radicals [73]. The hydroxyl and methoxyl functional groups present in this macromolecule can donate hydrogen to terminate oxidation propagation reactions [73,74]. The antioxidants help control the excessive production of reactive oxygen species, strongly associated with severe inflammation and lead to chronic conditions. Antioxidant activity of PAN/AgNPs was evaluated by DPPH free radical scavenging in vitro. The phenolic content of alkali lignin scavenges the free radicals from DPPH; thus, the purple color of the DPPH solution turns yellow. The colorimetric difference of diphenyl-picrylhydrazine radical solution was estimated via a UV-visible spectrophotometer. The antioxidant efficiency of PAN/AgNPs is shown in Figure 11. In PAN/AgNPs, a regular increase in the radical scavenging activity can be observed with the increased time and amount of alkali lignin used for AgNPs synthesis. PAN/AgNPs1, PAN/AgNPs3, and PAN/AgNPs5 showed maximum antioxidant activity of 67.4 ± 0.14, 67.4 ± 0.08, and 67.4 ± 0.23 at 12 h of time interval, whereas PAN/AgNPs2, PAN/AgNPs4, and PAN/AgNPs6 showed maximum antioxidant activity of 78.3 ± 0.12, 77.37 ± 2.42, and 78.37 ± 0.12% at 12 h of time interval. The concentrations of lignin used in AgNPs synthesis have a significant effect on the antioxidant activity of the membranes (*P*-value = 0.00, R^2^ = 99.99). Within 2 h of incubation, the antioxidant activity of PAN/AgNPs reached ⁓69%. The instantaneous free radical scavenging behavior of lignin also supports earlier published works [75]. The more the immersion time of nanofibrous in the DPPH, the higher was the radical scavenging activity by lignin, thus increased discoloration of the DPPH solution. Thus, lignin cover on the nanofiber surface presented an added advantage of the antioxidant feature to PAN/AgNPs.

### 3.11. Practical Applications and Future Research Perspective

The outstanding properties of polymeric nanofibers widened their application in a variety of fields. Our prepared Ag composite PAN nanofibers were targeted for effective antibacterial activity against both Gram-negative and Gram-positive bacterial strains, along with the efficient antioxidant activity of surface coated lignin. The enhanced surface wetting properties, release behavior of Ag elements, and the free radical scavenging property of PAN/AgNPs were suitable for the treatment of acute wounds. The efficient antibacterial activity of PAN/AgNPs also makes them suitable for the antibacterial filtration membrane.

The utilization of lignin as a green material for the synthesis of AgNPs coated polymeric nanofibers is relatively new and still needs a lot of research owing to the complex structure of lignin. Further investigations on the in vitro and in vivo studies should be conducted for examining the biocompatibility of lignin-induced PAN/AgNPs system.

## 4. Conclusions

Herein, we reported an antibacterial electrospun PAN nanofibrous membrane, which was surface coated with lignin synthesized AgNPs. SEM observations revealed smooth and beadless morphology of the nanofibers with slight increase in the average diameters of the nanofibers. TEM micrographs and ICP spectrometry revealed that the amount of formed AgNPs is proportional to both precursor salt and alkali lignin concentration. XPS analyses confirmed the presence of AgNPs on the surface of nanofibers. In addition to synthesizing AgNPs, the observed lignin layer on nanofibers’ surface presented multifunctional performance to PAN/AgNPs. The hydrophobic PAN nanofibers exhibited hydrophilicity and good liquid absorption property. Free radical scavenging capability was observed for all PAN/AgNPs resulting from the surface coated lignin. The resultant composite nanofibers showed excellent antibacterial activity against Gram-positive *S. aureus* and Gram-negative *E. coli* bacterial strains. The metal release amounts were observed to be proportionally associated with the loaded Ag contents with initial burst release of Ag elements within 24 h.

## Figures and Tables

**Table 1:** Sample coding.

AgNO_3_ Concentration	Alkali Lignin Amount	Sample Code
5 mM	2 g/L	PAN/AgNPs1
	4 g/L	PAN/AgNPs2
10mM	2 g/L	PAN/AgNPs3
	4 g/L	PAN/AgNPs4
15 mM	2 g/L	PAN/AgNPs5
	4 g/L	PAN/AgNPs6

**Table 2:** Thickness of the membranes, average diameter of nanofibers, and average diameter of AgNPs.

Sample Code	Thickness of the Membrane (mm)	Average Diameter of Nanofibers (nm)	Average Diameter of AgNPs (nm)
PAN nanofiber	0.014	512 ± 73	‒ ‒ ‒ ‒
PAN/AgNPs1	0.06	604 ± 39	9 ± 2.3
PAN/AgNPs2	0.06	614 ± 46	14 ± 3.0
PAN/AgNPs3	0.04	605 ± 59	14 ± 2.6
PAN/AgNPs4	0.02	642 ± 82	15 ± 3.9
PAN/AgNPs5	0.13	665 ± 86	16 ± 4.2
PAN/AgNPs6	0.05	673 ± 98	12 ± 2.6

## Data Availability

The data presented in this study are available on request from the corresponding author. There is no public repository at our institution.

## Abbreviations

PAN—Polyacrylonitrile, AgNPs—Silver nanoparticles, PAN/AgNPs—PAN nanofibers surface-functionalized with silver nanoparticles, WCA—Water Contact Angle, DPPH- 1, 1-diphenyl-2-picryl hydrazyl, SEM—Scanning Electron Microscope, TEM—Transmission Electron Microscope, FTIR-ATR—Fourier Transform Infrared-Attenuated Total Reflectance, WAXRD—Wide Angle X-ray Diffraction, XPS—X-ray Photoelectron Spectroscopy.
